# Scaffolds from Surgically Removed Kidneys as a Potential Source of Organ Transplantation

**DOI:** 10.1155/2015/325029

**Published:** 2015-02-10

**Authors:** Marek Karczewski, Tomasz Malkiewicz

**Affiliations:** ^1^Department of Transplantology, General, Vascular and Plastic Surgery, Clinical Hospital of Poznan University of Medical Sciences, 60-780 Poznan, Poland; ^2^Clinical Hospital of Poznan University of Medical Sciences, 60-780 Poznan, Poland

## Abstract

End stage renal disease (ESRD) is a common disease, which relates to nearly 600 million people in the total population. What is more, it seems to be a crucial problem from the epidemiological point of view. These facts lead to a further necessity of renal replacement therapy development connected with rising expenditures for the health care system. The aim of kidney tissue engineering is to develop and innovate methods of obtaining renal extracellular matrix (ECM) scaffolds derived from kidney decellularization. Recently, progress has been made towards developing a functional kidney graft *in vitro* on demand. In fact, decellularized tissues constitute ideal natural scaffolds, due to the preservation of native ECM architecture, as well as of cell-ECM binding domains critical in promoting cell attachment, migration, and proliferation. One of the potential sources of the natural scaffolds is the kidney, which cannot be transplanted immediately after excision.

## 1. Background

The number of patients suffering from end stage renal disease (ESRD) is rising every year. The main causes of kidney failure patients include glomerulonephritis, diabetic kidney disease, hypertensive nephropathy, polycystic kidney disease, Alport syndrome, and other factors, which gradually decrease the glomerular filtration rate. Pharmacological nephroprotection has turned out to be ineffective in the long run; therefore, it is necessary to employ renal replacement therapy (RRT), usually haemodialysis (HD). The dialysed patients still present high morbidity rates in terms of comorbidities, as well as high mortality rates. Filtration during haemodialysis is not entirely physiological due to membrane filtration restrictions resulting from thickness, shapes, and the size of the pores, as well as from occlusions with protein deposits and blood clotting. Moreover, haemodialysis makes the lives of the patients suboptimal and generates high costs of treatment. What is necessary to raise the patients' quality of life is an improvement of the filtration structure of membranes and the refinement of filtration parameters. However, the role of kidneys is not limited to mechanical filtration and blood cleansing, but also involves hormone secretion, red blood cells production, and bone metabolism through calcium-phosphorus balance. Thus, the optimum treatment would be to replace the disabled organ with a healthy kidney.

The first successful kidney transplants date back to 1954 when in Boston (USA) Joseph Murray and John Merrill together with their team managed to successfully transplant a kidney between monozygotic twins without immunosuppressant treatment. The surgery made it possible to transplant kidneys as a way to manage chronic kidney failure and shifted organ transplantation from the area of experimental treatment to a clinical method. Immunological discoveries, the introduction of new medications, and the improvement of the immunosuppressive protocols have led to a further development of transplantology. What is more, all these factors have also prolonged the lifespan of organs and their recipients. Excellent results of transplants have been reflected in the demand for the transplant organs, which is significantly higher than the available supplies. The donor pools have been expanded to include living donors, related and unrelated ones, pair exchange, and the expansion of marginal kidney donor criteria. However, all of this is still insufficient in comparison to the number of recipients awaiting the transplant. The mortality rate in the group of chronically dialysed patients awaiting the transplant is much higher than in the patient group after transplant surgery, even if the so-called suboptimal organs were employed. Taking into account all of the abovementioned information, there exists a need for an inexhaustible source of transplant organs.

One of the potential sources is the kidneys which cannot be transplanted immediately after excision due to anatomical anomalies, advanced glomerulosclerosis, tubular atrophy, interstitial fibrosis, renal vessel disease, necrosis of the renal cortex, as well as factors exceeding the allowed time of cold renal ischaemia, lengthy duration of warm ischaemia, and renal function insufficiency [1]. According to a growing number of scientists, there is a way to use such organs for transplantation by means of bioengineering and regeneration [2]. In recent years many groups of researchers have claimed to have used autologous cells for reconstruction of relatively simple organs, such as vessels, the urinary bladder, the upper airways, the oesophagus, and the urethra, which were transplanted to patients with acceptable organ function with a short and medium postoperative time period [3–6]. Due to a relatively simple structure of these organs, oxygen and nutrients demand first takes place by diffusion, which allows neoangiogenesis to develop gradually. Tissue transplant bioengineering is based on the idea of employing scaffolds, both natural and artificial, constituting a three-dimensional scaffolding on which cells settle, seed, and differentiate into a new organ. In organ regeneration, biological materials can be used as scaffolds (e.g., proteins, peptides, and bicarbonate), as well as synthetic materials (e.g., polymers, metals, and pottery), and animal or human tissues. The choice of the material needs to provide for biological and mechanical properties necessary for the organs formed* in vivo* to function [7]. Up to date, in* de novo* kidney reconstruction hollow fibres, thermoactive and biodegradable polymers, and collagen have been used [8]. However, both synthetic and natural polymers are unable to recreate the complex structure of a functional kidney.

## 2. The Renal Extracellular Matrix

It would seem that the best solution in obtaining an* ex vivo* organ is the scaffold from a cell-free organ maintaining the features of the extracellular matrix (ECM) based on the support of the natural architecture of an organ and taking part in the settlement, proliferation, and migration of cells. An attempt to recreate a human kidney* in vitro* by means of a scaffold constitutes an extremely difficult challenge. It is mainly due to the fact that there is a necessity to combine cells of various kinds with circulation and microcirculation, which are indispensable for the proper functioning of the kidney. In terms of solid organs, as it is the case with kidneys, it is impossible to adopt an organ without including the vascular system of a transplanted organ into the cardiovascular system of the recipient. In fact, the key role in obtaining normal functioning of any solid organ is recreating a vascular supply of 1–3 mm.

The scaffold of a kidney, made from the extracellular matrix, has the necessary potential to be included in the recipient's cardiovascular system owing to the internal biocompatibility, the architecture, and the cardiovascular structure. In addition, such scaffolds have the potential to differentiate the progenitor cells into organ-specific phenotypes [9].

Research in the areas of solid organ bioengineering using cell-on-scaffold seeding technology conducted in recent years has been promising [10–14]. Cell-on-scaffold seeding technology is based on the theory that cells remain alive, seed, and maintain their function due to the structural support. In an* in vitro* situation such a role can be attributed to the extracellular matrix [15, 16]. The decellularized extracellular matrix structure not only is able to keep the morphological shape of an organ, but also maintains the macro- and microarchitecture of the renal parenchyma. In order to maintain viability and functionality of the made organ it is necessary for the ECM to play the key role in the biomolecular structure, as well as in the cellular adhesion, signalling processes, binding growth factors, and the implantation of the cells by means of the vascular network skeleton. It is crucial for the biocompatibility of such an organ to dispose of the crucial tissue typing reagents, for example, HLA classes I and II [2] in the decellularization process. Obtaining an organ structure without antigen tissue typing but with a possibility of its implantation by the autologous cells may lead to the conclusion that using immunosuppression is unnecessary and thus suggests tolerance [17]. Although there are doubts regarding the quality of the organ when biomaterial has been obtained from disqualified transplants, the conducted research demonstrates reversibility of the changes [18].

## 3. Decellularization

Techniques of solid organ decellularization require more refined methods due to the thickness of an organ and its complex internal structure. Thus, it was necessary to develop techniques of decellularizing the organ structure and removing the residual DNA, preserving the extracellular matrix integrity of an organ. In fact, if based on the flow-through technique, the vascular structure of an organ remains intact and may be used as a matrix for new interstitial or progenitor cells. The preparation of the structure of an organ requires gathering proper reagents not to break the vascular network skeleton. There are a number of factors, which may influence the process of organ decellularization, such as cellular density, organ density, fat contents, the thickness of an organ, and the properties of the substances removing the cells [11]. In addition, it is crucial to select appropriate mechanical forces in this process, for example, pressure, the flow of the decellularization factors, as well as flow-through techniques, and the backflow to an organ. It is perfusion and decellularization detergents which allow for the removal of the cellular residue through the vascular system. There are different decellularization strategies using a perfusion of detergents or enzyme solutions through the renal vasculature (Ross et al. 3% Triton X-100, DNase, 4% SDS: time not specified [11], Song et al. 1% SDS: 12 h; 1% Triton X-100: 30 min [13], Burgkart et al. 0,66% SDS: 1 h [19], Bonandrini et al. 1% SDS: 17 h [20], Sullivan et al. 0,5% SDS: 36 h, DNase: overnight [21], Nakayama et al. 1% SDS: 7–10 days [22], O'Neill et al. 0.02% trypsin: 2 h; 3% Tween: 2 h; 4% sodium deoxycholate: 2 h [23]).

Due to different tissue properties, such as cellularity, density, thickness, and lipid content, the need to alter ECM composition and to avoid ultrastructure disruption establishing the decellularization protocol constitutes a critical determinant of the clinical success. Many decellularization techniques have been established for rodent kidneys; however, the methods for murine kidney would not be applicable in a clinically relevant sized kidney, such as sheep or pigs. Since porcine kidney is the most common source of clinical xenografts, efficient decellularization protocols for organs of this size must be established in order to enable clearance of all native cellular components, as well as to preserve ECM in a clinically relevant time [21, 24–26]. During the decellularization process, detergents act on different tissues in different patterns [27]. Two detergents are successfully used in the process of whole organ decellularization: nonionic Triton X-100, at concentration of 1%, and an ionic detergent of sodium dodecyl sulfate (SDS) in concentrations from 0,1 to 5% [21, 28–30]. The size of the organ itself is not critical for the decellularization process, provided vascular integrity is maintained [21, 30]. The process of decellularization leads to the cell mediated functions loss, such as solute transport and macromolecule sieving owing to the loss of filtration and reabsorption properties across glomerular and basement membranes [13].

## 4. Sterilization of ECM Scaffolds 

Before* in vitro* or* in vivo* use, biological ECM scaffolds had to be sterilized to remove all endotoxins, possible viral and bacterial DNA which may be present. The technique used in the process may be simple incubation in solvents or acids [31–33]. The disadvantages of these methods stem from insufficient penetration and possible damage of the ECM construction [34]. What is more, gamma irradiation, electron beam irradiation, or ethylene oxide exposure alters ultrastructure of the ECM scaffold not only in terms of mechanical properties. In addition, by exciting the host immune response ECM proper function impairment after implantation may occur [35, 36]. In fact, supercritical carbon dioxide inactivates bacterial spores and viruses in multilog progress with minimal changes in mechanical properties of the ECM [37].

## 5. Decellularized Tissue Characterisation

The ultimate goal of decellularization protocols is the removal of all cellular components without affecting the extracellular composition, mechanical integrity, and biological activity. It is worth noting that both the histological and quantitative analyses of ECM show the preservation of collagen and glycosaminoglycans. What is more, immunochemistry in the decellularized scaffolds presents the expression of most common ECM proteins, such as collagens, laminin, and heparan sulfate proteoglycans. No decellularization technique is able to remove 100% of native cell material, which may contribute to cytocompatibility problems* in vitro* and adverse host response upon* in vivo* reintroduction of cells [38, 39]. The threshold concentration of residual cellular components within ECM, which is sufficient to elicit host response, depends on the ECM source, host immune function, and tissue environment into which the ECM is implanted. The minimal satisfying criteria for the decellularization process in this aspect are <50 ng dsDNA per mg ECM dry weight <200 bp DNA fragment length, lack of visible nuclear material in tissue sections stained with 4′,6-diamidino-2-phenylindole (DAPI) or H&E [40]. There is no consensus regarding the influence of different reagents on the mechanical properties of the ECM scaffold. Although detergents may disrupt collagen, thereby decreasing mechanical strength of tissue, the effect is different even in similar tissues [41, 42].

## 6. Recellularization of Acellular Scaffolds

A kidney consists of approximately 30 different specialized cell types, 2 million glomeruli essential for renal filtration process, and well-developed capillary system [43]. For transplant purposes, two major compartments must be repopulated: the parenchymal compartment for organ-specific function as well as the endothelium to allow the necessary implantation and perfusion. Recellularization of the ECM scaffolds is definitely the most complex phase. No study thus far has provided sufficient results of renal scaffold repopulation and differentiation with adequate cells, which would enable artificial organ transplantation. Hence, many efforts are focused on the search of the best cell source and the optimal growth conditions of possible repopulating cells.

## 7. Adult Stem Cells

A subset of renal adult stem cells with the expression of stem cell markers CD24, CD133, CD 146, and Pax-2 have been found in Bowman's capsule, renal papilla, glomeruli, pericytes, and proximal tubules [44–48]. Humphreys presents tubular epithelial cells as the main source of regeneration after ischaemic injury [49]. Moreover, distal tubular cells are mostly involved in cytokines and growth factor production, such as EGF, IGF-1, and hepatocyte growth factor [49, 50]. Human nephron progenitor cells (hNPCs) express NCAM1^+^ and possess high clonogenic potential to repair a chronically injured kidney and regenerate its structure [51]. In addition, human adult kidney epithelial cells (hKEpCs) expressing NCAM1^+^are involved in the experimental production of the epithelial renal tissue [52]. Isolation and* in vivo* clonal analysis of progenitor cells from tubules and glomeruli make it possible to mark cells directly involved in kidney regeneration [53].

## 8. Bone-Marrow Stem Cells

Two major populations of bone-marrow stem cells, hematopoietic stem cells (HSCs) and mesenchymal stromal cells (MSC), could be considered for kidney cell replacement; however, this fact is still far from clinical relevance [54, 55].

## 9. Mesenchymal Stem Cells

This kind of cells can be isolated from different tissues and have multilineage differentiation potential as well as the ability to migrate to the injured area of the kidney. MSCs activate lymphocytes T and B and stimulate cytokines, TGF-*β*, hepatocyte, fibroblast, and endothelial growth factors [56, 57]. What is more, adipocyte derived stem cells (ADSC) constitute an abundant source of mesenchymal stem cells, are easy to harvest, and possess the property of stimulating angiogenesis. Many studies revealed that injection of ADSCs into experimentally injured kidney reduced fibrosis, attenuated the deterioration of renal function, and stimulated neoangiogenesis [58, 59].

## 10. Embryonic Stem Cells

These cells are derived from blastocysts and possess pluripotent properties. Furthermore, they have the capability of differentiation into tissues derived from the three germ layers. Renal progenitor cells derived from ESCs are able to differentiate into glomerular-like structures, and after implantation into kidney they become integrated into renal proximal tubules [60, 61]. Undifferentiated or partially developed kidney precursor cells can be also obtained from early embryos and fetal tissue. Successful organogenesis was achieved by transplantation of early progenitors with mesenchymal cells and ureteric bud branches [62]. ESCs are associated with uncontrolled growth and teratoma formation, as well as the extraction of these cells which involves destruction of embryos, thus leading to ethical issues [62].

## 11. Stem Cells Derived from Placenta and Amniotic Fluid

Human amniotic stem cells (HASCs) have a self-renewal potential and multilineage differentiation capacity. In comparison with ESCs, HASCs express transcription factors and surface markers: octamer-binding transcription factor 4 (OCT-4) and stage specific embryonic antigen (SSEA-4) [63]. After injection HASCs integrated into metanephric structures leading to recovery in kidneys with acute tubular necrosis [64, 65].

## 12. Cloned Renal Cells

Cloning technique in a bovine model based on microinjection of skin fibroblast nucleus into an enucleated oocyte was first described by Lanza et al. [66]. After seeding into a biodegradable scaffold they were able to filtrate, reabsorb, and secrete. Similar to methods involving ESCs, this technique is associated with ethical controversy.

## 13. Induced Pluripotent Stem Cells

The technique of pluripotent stem cells (iPSCs) induction, involving reprogramming human fibroblasts to pluripotent stem cells by the addition of gens,* Oct3/4, Sox2, c-Myc,* and* Klf4*, was first described by Takahashi and Yamanaka [67]. Different types of adult stem cells may have distinct critical factors to be reprogrammed. The use of iPSCs does not involve ethical issues. However, on the other hand, the risk of uncontrolled growth is increased by the addition of oncogens to be reprogrammed [68]. iPSCs can be generated from different sources like mesangial and epithelial cells from urine, podocytes, and proximal tubular cells which provides promising results for clinical use [66, 68]. Human PSCs (hPSCs), which have the ability to differentiate into multiple cells, possess tremendous potential in regenerative medicine. Nonetheless, limited success has been achieved in terms of the kidney-related cells generation. In order to achieve kidney formation, reciprocal interaction between metanephric mesenchyme and ureteric bud is necessary [69].* In vitro* derived kidney should contain single collecting duct tree draining to ureter and needs to be properly vascularized for adequate filtration function. To accomplish this task it is crucial to generate large quantities of a single differentiate cell type, or to generate self-organizing nephron-like structures. He, first report of the differentiation of iPSCs into nephrogenic intermediate mesoderm was submitted by Osafune group. However, these cells could not form ureteric bud tissue [70]. Since then, several protocols demonstrating successful derivation of kidney-related cell lineages from hPSCs and 3D kidney formation were published [71, 72]. One group involved a three-step protocol for synchronous generation of ureteric bud and metanephric mesenchyme from hPSCs. Wnt signalling was used for differentiation of ESCs into mesoderm and FGF9 signalling for intermediate mesoderm generation. In the third step, both BMP7 and retinoic acid were used for metanephric mesenchyme and ureteric bud formation [73]. For total recapitulation of kidney development, three types of progenitors are required: metanephric mesenchyme progenitors, ureteric bud progenitors, and angioblasts. Nevertheless, the question remains as to if they could assemble correctly into a functional kidney after the generation of all progenitors from hPSCs.

## 14. Vascularization

Another vital challenge is achieving full vascularisation of the solid organ. The fact that oxygen transport by diffusion is possible only within a small radius from the source of it leads to a situation where the oxygen access is a limiting factor for the used scaffold [74]. Hence, solid organs with bigger structure area, such as the kidneys, the liver, or the heart, require undertaking a full revascularization function. Such an effect may be obtained by means of employing angiogenic factors and prevascularization at both the* in vitro* and* in vivo* stages [75]. Therefore, the employment of scaffolds from decellularized organs is useful because of the preservation of the extracellular matrix (ECM) crucial in the process of adhesion and proliferation. Moreover, the use of such scaffolds leaves the vascular network intact and makes it possible to cover them with the vascular endothelium during the process of settling the cellular lines [40, 76]. Using decellularized scaffolds with the simultaneous employment of angiogenic factors, such as vascular endothelial growth factor (VEGF), increases revascularization ability. However, the incubation period lasting from a few days up to a few weeks is necessary for full perfusion to take place [40]. An* in vitro* prevascularized scaffold does not provide an instantaneous vascularisation of an organ. In order to obtain it, it is necessary to connect the scaffold's vascular skeleton with the newly made vessels of the host. In fact, an* in vivo* prevascularization may provide a constant blood flow by means of an instantaneous connection with the donor's vascular system. It requires a period of spontaneous preimplantation angiogenesis during which degenerative changes in an organ take place because of the nutritious limitations. The direct connection of the vascular system with the recipient's vessels accompanied by angiogenesis stimulating factors is a better solution comparing both revascularization techniques [77]. The creation of mature and stable capillaries, as well as larger vessels, depends on the pace of endothelial cells proliferation. Therefore, the first step after the connection of the scaffold with the vascular system of the recipient is a quick recreation of not only the endothelial cells, but also other cells building the vascular wall, such as pericytes and vascular smooth muscles. Repopulation with the use of these cells is a marker of the revascularization process and ensures a stable vascular system of the formed organ [77]. Taking all of the abovementioned factors into account, it would seem feasible to obtain a properly functioning organ based on a three-dimensional scaffold, stimulated by angiogenic factors in the preimplantation period and seeded with endothelial cells and pericytes [78].

## 15. Bioreactors

In order to obtain a three-dimensional solid organ, such as the kidney, it is essential to provide the seeding cells with conditions closely resembling the physiological ones. In terms of organ bioengineering, perfusion bioreactors are most commonly used for the recreation of the vascular system and the appropriate scaffold seeding to take place [79]. It allows for the administration of the seeding cells through the vascular system of an organ stored in the bioreactor, which maintains the optimum conditions for the cell growth [80]. The bioreactor with a flow similar to that of a physiological one (pulsing or continuous) in which the regulation of pressure and flux is possible and allows for optimum conditions particular seeding cells. Each of the organs created in the bioreactor requires additional conditions resembling the physiological environment of an organ. For instance, lung bioreactors must provide the environment for perfusion and ventilation, whereas heart bioreactors need to supply physiological flow and pressure, and provide the electrical stimulation in order to obtain the contractility of the cells [81–83]. In order to provide both the test-retest reliability and the possibility of obtaining organs on a larger scale, bioreactors need to ensure the precision of control over the environmental factors and provide the regulation of PH, pressure, oxygen contents, and nutrients supply [84].

## 16. Transplantation of Regenerated Kidneys

In the rat model, partial reabsorption of glucose and electrolyte was restored after the transplantation of regenerated kidneys, indicating engraftment and function of podocytes and endothelial and tubular epithelial cells [13]. Neither bleeding nor graft thrombosis was observed during perfusion through transplanted kidney vasculature [13]. Compared to deceased donor kidneys, glomerular filtration rate was lower and depended on renal perfusion pressures. Therefore, taking into account the abovementioned facts, bioengineered kidneys could become a fully implantable source and a treatment option for end stage renal disease.

## 17. Scaffold Sourcing

To obtain organs by means of bioengineering it is crucial to select the proper scaffold source. Human kidneys are already mentioned source, which due to a variety of reasons are removed or disqualified for transplant. Such organs seem to be the perfect source due to their specific distinction in terms of the properties of the extracellular matrix. The issue is that because these organs are rejected for transplantation, there is possibility of organ damage. Because of the similarity in the organ size and its microarchitecture, it seems that pig or sheep organs constitute a suitable source. It is possible to obtain ECM organ scaffolds from animal sources, which can later be seeded with human cells, and then use them in transplant surgery [85]. Protocols for rapid decellularization to enable clearance of native cellular components with preservation of ECM would be required for clinical use. Sullivan group constructed and tested a high-throughput system of multiple-whole-organ decellularization, while maintaining a consistent outcome, and provides flexibility to switch between different decellularization protocols [21].

## 18. Conclusions and Future Directions

Bioengineering poses a number of questions and ambiguities. The selection of a suitable source of organs, standardization of scaffold obtaining and seeding them with cells, achieving proper organ revascularization, and their storage constitute the most crucial challenges. In addition, there are a number of ethical and legal issues. However, bearing in mind the length of the transplant waiting lists globally, which stem from the lack of donors, there is a growing need for an alternative source of organs. The cell-scaffold technology constitutes one of the possible alternative ways to treat patients suffering from end stage renal disease. In fact, this method may prove superior to the current methods as it makes it possible to achieve tolerance of the artificially grown organ [24, 85–87]. In terms of bioengineering, it is crucial to understand the developmental processes of the organs and comprehend the mechanisms behind a number of organ pathologies and ways of treating them. In fact, many regenerative centers worldwide continue working to provide a reliable source of scaffolds and to find feasible strategies in order to develop bioengineered kidneys [46].
